# Evaluation of the Efficacy of 50% Autologous Serum Eye Drops in Different Ocular Surface Pathologies

**DOI:** 10.1155/2014/826970

**Published:** 2014-07-22

**Authors:** Francesco Semeraro, Eliana Forbice, Osvaldo Braga, Alessandro Bova, Attilio Di Salvatore, Claudio Azzolini

**Affiliations:** ^1^Eye Clinic, Department of Neurological and Vision Sciences, University of Brescia, Piazzale Spedale Civili 1, 25123 Brescia, Italy; ^2^Department of Surgical and Morphological Sciences, Section of Ophthalmology, University of Insubria, Ospedale di Circolo, Via F. Guicciardini 9, 21100 Varese, Italy

## Abstract

*Purpose*. This study evaluated the efficacy of 50% autologous serum eye drops in ocular surface diseases not improved by conventional therapy. *Methods*. We analyzed two groups: (1) acute eye pathologies (e.g., chemical burns) and (2) chronic eye pathologies (e.g., recurrent corneal erosion, neurotropic keratitis, and keratoconjunctivitis sicca). The patients were treated for surface instability after conventional therapy. The patients received therapy 5 times a day until stabilization of the framework; they then reduced therapy to 3 times a day for at least 3 months. We analyzed the best corrected visual acuity, epithelial defects, inflammation, corneal opacity, and corneal neovascularization. We also analyzed symptoms such as tearing, burning, sense of foreign body or sand, photophobia, blurred vision, and difficulty opening the eyelids. *Results*. We enrolled 15 eyes in group 1 and 11 eyes in group 2. The average therapy period was 16 ± 5.86 weeks in group 1 and 30.54 ± 20.33 weeks in group 2. The epithelial defects all resolved. Signs and symptoms improved in both groups. In group 2, the defect recurred after the suspension of therapy in 2 (18%) patients; in group 1, no defects recurred. *Conclusions*. Autologous serum eye drops effectively stabilize and improve signs and symptoms in eyes previously treated with conventional therapy.

## 1. Introduction

The ocular surface is a morphofunctional unit that owes its action to the perfect cooperation of all its structures (i.e., conjunctival and corneal epithelium, lacrimal apparatus, and eyelids) [1]. In particular, tears are important in maintaining the stability of the ocular surface because of its lubricant, mechanical, epitheliotropic, and antimicrobial functions [2]. A qualitative and quantitative deficiency of tears can lead to the persistent and progressive damage of the ocular surface with a compromised wound-healing process [3–5]. In this situation, the conventional therapeutic options are intensive artificial tears, punctual occlusion, contact lenses, and appropriate management of adnexa diseases [2]. However, these therapies are limited in supplying the neurotrophic factors, vitamins, and immunoglobulins necessary for the health of the ocular surface [2]. Just with this target arises the use of autologous serum (AS) eye drops. In 1970, the use of AS for treating ocular surface disorders began when it was used to treat ocular alkali burns [6]. However, only later—first with Fox et al. [7] and then with Tsubota et al. [8]—did this therapy enter clinical practice for the treatment of different ocular surface diseases. Since its introduction, this treatment has become increasingly popular and the indications for its use have expanded rapidly [9]. To date, AS is used for the treatment of persistent epithelial defects [10–15], dry eyes [1, 8, 16–27], neurotrophic keratopathy [28], recurrent erosion syndrome [29, 30], superior limbic keratoconjunctivitis [31], and chemical injuries [32, 33].

The rationale for the use of AS arises from its strong similarity to tears, which contains growth factors, cytokines, vitamins, and bactericidal components that provide the necessary nutritional factors to maintain cellular tropism and reduce the risk of contamination and infection during epithelial repair processes [2]. In fact, human serum contains substances such as epithelial growth factor (EGF), which speeds epithelial cell migration and has antiapoptotic effects [34]; transforming growth factor *β* (TGF-*β*), which is involved in the epithelial and stromal repair process [35]; vitamin A, which seems to prevent epithelium squamous metaplasia [36] and modulates the expression of thrombospondin 1 (TSP1) [37], thrombospondin 2 (TSP2), vascular endothelial growth factor A (VEGF-A), metallopeptidase 9, and TGF-*β* to promote wound healing [38]; albumin, which has antiapoptotic activity [39]; *α*-2 macroglobulin, which exhibits anticollagenase activity; and fibronectin, which is important in cell migration [11, 40]. Autologous serum also contains neuronal factors such as substance P (SP) and insulin-like growth factor 1 (IGF-1), which seem to have a role in corneal epithelium migration and adhesion [41]. In addition, AS contains immunoglobulins (Ig) such as IgG and IgA and lysozyme that provide bactericidal and bacteriostatic effects [2, 42]. Furthermore, AS is superior to artificial tears in maintaining corneal epithelium health because it is free of preservatives [43] (which potentially induce toxic or allergic reactions [44]) and its osmolality and biomechanical properties are similar to those of natural tears. The aim of this study was to evaluate the efficacy of 50% autologous serum eye drops in the treatment of symptoms and objective signs in different ocular surface diseases that are not improved by conventional therapy.

## 2. Materials and Methods

This single-center prospective study was conducted from January 2008 to January 2013. We enrolled patients who came to our department because of ocular surface dysfunction related to trophic deficiency (e.g., recurrent corneal erosion, neurotrophic keratitis, and keratoconjunctivitis sicca [Sjögren and non-Sjögren-related]) and chemical burns.  Table 1 lists the inclusion and exclusion criteria of the study.

Written informed consent was obtained from all subjects before their enrollment after they received an explanation of the nature and possible consequences of the study. The institutional review board of the Spedali Civili Hospital (Brescia, Italy) ethics committee approved the study. The Declaration of Helsinki was followed. All patients had a screening visit. The best corrected visual acuity (BCVA) was measured using a Snellen-type acuity in all patients.

Specialized ophthalmologists collected clinical data by slit-lamp examination (using corneal fluorescein staining to study epithelial defects) and evaluated epithelial defects, inflammation, corneal opacity, and corneal neovascularization. A grading scale of 1 to 3 was used to identify the severity of these signs in which grade 1 was “absence of signs”; grade 2 was “slight”; and grade 3 was “severe.”

A similar scale was used for subjective symptoms. We recorded data on tearing, burning, sense of foreign body or sand, photophobia, blurred vision, and difficulty in opening the eyelids on a scale graded from 1 to 4 in which grade 1 was “no symptom,” grade 2 was “slight,” grade 3 was “moderate,” and grade 4 was “severe.” When possible, a picture of the anterior segment of the eye was obtained.

Patients were divided into 2 groups: (1) patients with an acute illness (e.g., chemical burns) and (2) patients with chronic disease (e.g., recurrent corneal erosion, neurotropic keratitis, and keratoconjunctivitis sicca (non-Sjögren and Sjögren-related)).

Group 1 eyes had chemical burns [grades II and III chemical injuries (based on the Dua classification [45])]. We treated the eyes with first-aid therapy, which included irrigation with normal saline or glucose to normalize the ocular surface pH, topical anti-inflammatory drugs, and antibiotic with eye bandage or contact lens. The treatment with AS eye drops was initiated after an average of 7 days of corticosteroid therapy in patients with persistent inflammation, epithelial defects, or any type of ocular surface instability without significant stem cell deficiency (i.e., more than one quadrant). The group was treated 5–6 times per day for 1 month in association with anti-inflammatory therapy (which was progressively decreased during the month) and then was reduced to 3 times daily until the absence of symptoms for at least 3 months without support therapy.

Group 2 included patients with chronic diseases that were unresponsive to conventional therapy (e.g., lubricating drops and ointments, punctal plug, bandage contact lenses, tarsorrhaphy, and gold eyelid weight). The treatment in this group was 5 times a day for 3 months and then was reduced progressively to 3 times a day for 3 months until the absence of symptoms. An antibiotic (moxifloxacin/netilmicin/tobramycin) was administered 4 times a day until the closing of the epithelial defect. No other supportive therapies were used during the treatment period. For each patient, the specialist could modify the therapy whenever necessary. The change was registered and justified.

Autologous serum drops were produced in the following manner. A total of 200 mL of blood was procured by venipuncture and collected in a sterile container. The blood was allowed to stand for 24–48 h at 4°C to allow clotting. The blood was centrifuged at 4000 rpm for 10 min. The serum was separated from the blood and diluted with saline to 50% in a laminar flow cabinet. At this point, the product was quarantined until the outcome of the sterility test performed by the Laboratory of Microbiology of the AO Civil Hospital of Brescia (Brescia, Italy). The final product was formed from an average of 288 single-dose eye drops for venipuncture. Single-application packs were packed in bags of 20 and marked with a label plate.

At −30°C, the product is preserved for 6 months from the date of withdrawal. From the time of delivery, the product is stored at a destination (e.g., home freezer) at −20°C for a period not exceeding 3 months from the date of delivery and no later than the expiration date stated on the label. If the entire production process is successful, the eye drops are validated, which takes into account negative serology examinations, group control transmitted and validated by the Emonet management system (i.e., computer system with records of the personal data of patients and the procedure to be performed), and negative results of sterility control.

*Statistical Analysis*. The data were recorded on a predesigned pro forma and managed on a spreadsheet using the Microsoft Excel 2013 software (Microsoft Corp., Redmond, WA). All entries were checked for errors. Appropriate statistical tests were applied to analyze the results. The *t*-test was used to determine the significance of changes in subjective symptoms, BCVA, epithelial defects, corneal neovascularization, opacity, and inflammation before and after AS eye inoculation therapy in the 2 groups of patients. The significance (*p*) was defined as a probability of error <0.01.

## 3. Results

### 3.1. Patients

In this study 28 eyes of 28 patients were enrolled. Two patients dropped out of the study because of the impossibility of obtaining blood samples: the first patient was seropositive for the human immunodeficiency virus and the second patient did not have venous access.

Of the 26 eyes of the 26 patients treated with AS, 18 patients were men and 8 were women. The mean age was 39.6 ± 16.47 years (range, 19–81 years) in group 1 and 57.63 ± 16.59 years (range, 37–80 years) in group 2 (Table 2).

Group 1 consisted of 15 eyes (58% of all patients in the study) injured by chemical agents of different natures. Group 2 (i.e., patients with chronic eye diseases) consisted of 11 eyes as follows: 6 eyes with neurotrophic keratopathy, 3 eyes with keratoconjunctivitis sicca not Sjögren, and 2 eyes with keratoconjunctivitis sicca, Sjögren-related.

The average therapy period was 22.15 ± 15.44 weeks (range, 12–72 weeks), 16 ± 5.86 weeks in group 1 and 30.54 ± 20.33 weeks in group 2. Two patients, both in group 2, remain under treatment.

### 3.2. Clinical Data

The* visual acuity* in group 1 went from 2.4/10 ± 1.91/10 (mean ± standard deviation) to 6.25/10 ± 3.25/10 (*P* < 0.01) after treatment with an average gain of 4 Snellen lines. In group 2, visual acuity went from 2.96/10 ± 2.04/10 to 4.7/10 ± 3.37/10 (*P* < 0.01) with an average gain of 2 Snellen lines. The visual acuity improved in 100% of patients (Figures 3(a) and 3(b)). The 55% percent of patients with chronic eye diseases had associated eye pathologies affecting the final visual acuity such as maculopathy, cataracts, and diabetic retinopathy, whereas no acute patient had concomitant pathology. The signs that were evaluated in the clinical examination were the presence of epithelial defects, neovascularization, corneal opacity, and the degree of inflammation.

In group 1 before treatment, 100% of patients had inflammation (it extended to the entire eye in 73% of patients and was grade 2 in 27% of patients); 100% of patients had corneal opacity (grade 3 in 93% of patients and grade 2 in 7% of patients); and 60% of patients had neovascularization (grade 3 in 33% of patients and grade 2 in 27% of patients).

In group 2 before treatment, 100% of patients had corneal inflammation (grade 3 in 64% of patients and grade 2 in 36% of patients); 82% of patients had corneal opacity (grade 3 in 60% of patients and grade 2 in 40% of patients); and 55% of patients had corneal neovascularization (grade 3 in 36% of patients, grade 2 in 27% of patients, and grade 1 in 37% of patients) (Table 3).

*Evolution of the Clinical Data (Figure 2)*

*Epithelial Defects*. Epithelial defects were completely resolved (grade 1) by the end of followup in all patients. In 2 patients in group 2, the epithelial defect recurred after the suspension of therapy. One patient was administered a new cycle of AS eye drops that resolved the defect. In the second patient, a tarsorrhaphy was necessary owing to the severity of the ocular disease and the impossibility of restarting AS therapy because of the inability to perform blood sampling due to the patient's worsening condition.

*Inflammation.* After treatment in group 1, the inflammation was grade 1 (i.e., absent) in 87% of patients and grade 2 in 13% of patients with an improvement of 2 degrees in 58% of patients and 1 degree in 42% of patients. In group 2, inflammation was grade 1 (i.e., absent) in 81% of patients and grade 2 in 19% of patients with an improvement of 2 degrees in 55% of patients and 1 degree in 45% of patients. All patients improved. The average improvement was 1.60 ± 0.49 degrees in group 1 (*P* < 0.01) and 1.54 ± 0.50 degrees in group 2 (*P* < 0.01).

*Opacity.* After treatment in group 1, opacity became grade 1 in 60% of patients and grade 2 in the remaining 40% with an improvement of 2 degrees in 35% of patients and 1 degree in 54% of patients. In group 2, opacity became grade 1 in 55% of patients and grade 2 in 45% of patients. The improvement was 2 degrees in 18% of patients and 1 degree in 64% of patients; by contrast, 2 patients remained stable at grade 2 (these were diabetic patients with neurotrophic keratopathy).

The average improvement was 1.53 degrees ±0.52 in group 1 (*P* < 0.01) and 0.82 degrees ±0.60 in group 2 (*P* < 0.01).

*Neovascularization.* After treatment in group 1, neovascularization became grade 1 in 73% of patients and grade 2 in 27% of patients with an improvement of 2 degrees in 29% of patients and 1 degree in 64% of patients. In group 2, neovascularization became grade 1 in 73% of patients and grade 2 in 27% of patients with an improvement of 2 degrees in 27% of patients and 1 degree in 45% of patients, whereas there was no change in 1 diabetic patient with neurotrophic keratopathy. The average improvement was 1.11 degrees ±0.60 in group 1 (*P* < 0.01) and 1.17 degrees ±0.75 in group 2 (*P* < 0.01).

### 3.3. Symptoms

The symptoms reported in group 1 were (in decreasing frequency) burning, feeling a foreign body/sand in the eyes, tearing, photophobia, and blurred vision.

Tearing was present in 42% of patients at the first visit: 73% of patients had grade 4 and 27% of patients had grade 3.

Burning was present in 73% of patients at first visit: 79% of patients had grade 4, 11% of patients had grade 3, and 10% of patients had grade 2.

The sense of sand in the eyes was present in 78% of patients at the first visit: 55% of patients had grade 4 and 45% of patients had grade 3.

Photophobia was present in 65% of patients at the first visit: 53% of patients had grade 4, 37% of patients had grade 3, and 10% of patients had grade 2.

*Blurred vision* was present in 73% of patients at the first visit: 48% of patients had grade 4, 48% of patients had grade 3, and 4% of patients had grade 2.

Difficulty opening* the eyelids* was present in 42% of patients at the first visit: 64% of patients had grade 4, 27% of patients had grade 3, and 9% of patients had grade 2.

The most severe symptom was eye burning. The symptoms reported in group 2 (in decreasing frequency) were a sense of a foreign body in the eyes, blurred vision, burning, and photophobia. The feeling of sand in the eyes and blurred vision were the most severe symptoms (Table 3).

*Evolution of Symptoms*. Tearing at the end of followup was grade 1 in 55% of patients and grade 2 in 45% of patients. There was an improvement of 3 degrees in 36% of patients, 2 degrees in 55% of patients, and 1 degree in 9% of patients. All patients improved. The average improvement was 2.43 ± 0.49 degrees in group 1 (*P* < 0.01) and 2 ± 0.70 degrees in group 2 (*P* < 0.01).

Burning at the end of followup was grade 1 in 74% of patients, grade 2 in 21% of patients, and grade 3 in 5% of patients. There was an improvement of 3 degrees in 53% of patients, 2 degrees in 32% of patients, and 1 degree in 15% of patients (i.e., 100% of patients improved). The average improvement was 2.42 ± 0.76 degrees in group 1 (*P* < 0.01) and 2.28 ± 0.70 degrees in group 2 (*P* < 0.01).

The sense of sand in the eyes at the end of followup was grade 1 in 75% of patients, grade 2 in 15% of patients, and grade 3 in 10% of patients. There was an improvement of 3 degrees in 40% of patients, 2 degrees in 45% of patients, and 1 degree in 10% of patients; however, 1 (5%) patient with neurotrophic keratopathy showed no improvement. The average improvement was 2.45 ± 0.50 degrees in group 1 (*P* < 0.01) and 1.89 ± 0.99 degrees in group 2 (*P* < 0.01).

Photophobia at the end of followup was grade 1 in 71% of patients and grade 1 in 29% of patients.

There was an improvement of 3 degrees in 35% of patients, 2 degrees in 47% of patients, and 1 degree in 18% of patients (i.e., 100% of patients improved). The average improvement was 2.36 ± 0.77 degrees in group 1 (*P* < 0.01) and 1.83 ± 037 degrees in group 2 (*P* < 0.01).

Blurred vision at the end of followup was grade 1 in 63% of patients, grade 2 in 21% of patients, and grade 3 in 16% of patients. There was an improvement of 3 degrees in 32% of patients, 2 degrees in 37% of patients, 1 degree in 21% of patients, and no improvement in only 2 (10%) patients (the first patient had a chemical eye injury with a slight degree of injury and the second patient had Sjögren syndrome eye with a mild degree of injury). The average improvement was 1.91 ± 0.99 degrees in group 1 (*P* < 0.01) and 1.87 ± 0.92 degrees in group 2 (*P* < 0.01).

Difficulty opening* the eyelids* at the end of followup was grade 1 in 91% of patients and grade 2 in 9% of patients. There was an improvement of 3 degrees in 64% of patients, 2 degrees in 18% of patients, and 1 degree in 18% of patients (i.e., 100% of patients improved). The average improvement was 2.5 ± 0.76 degrees in group 1 (*P* < 0.01) and 2.25 ± 0.83 degrees in group 2 (*P* < 0.01).

## 4. Discussion

Autologous serum eye drops are actually used in the treatment of many ocular diseases, and many studies have demonstrated the effectiveness of AS eye drops in treating different conditions such as superior limbic keratoconjunctivitis [31], recurrent corneal erosion [12, 30], neurotropic keratopathy [10], and Sjogren's syndrome [8].

Despite clinical evidence of the efficacy of AS, a shared protocol for the preparation and the administration of this therapy is lacking because of the bureaucratic and technical difficulties of handling biological materials. The European Union (European Parliament and Council) has issued several directives concerning AS eye drops (1965/65, 1975/139, and 1975/318). However, in the European Union, individual countries regulate the manufacture and distribution of pharmaceuticals, and the use of serum eye drops remains an experimental approach [20]. The marketing authorization for a drug normally depends on proof of efficacy in clinical trials, implementation of quality control, reports of adverse effects, evidence of expert knowledge, and other regulatory issues. A doctor who makes or prescribes a specific medical product to treat a patient on a nominal basis is exempt from the requirement to obtain authorization as a “professional authorized by law to prescribe or administer drugs or devices so the responsibility in preparation and administration is entirely of the prescriber.” This has justified the limited use of AS eye drops [20, 46–48].

In addition, there have been few reports that show the efficacy of some blood products such as platelet-rich plasma (PRP) in treating chemical burns [32, 49]. Hence, we started using AS eye drops for different ocular surface diseases that were not responding to conventional therapies. Our study investigated a heterogeneous group of pathologies with different pathogeneses to understand better the role and potentialities of AS.

We chose to use 50% AS eye drops instead of other concentrations (e.g., 20% or 100%) used in some works in the literature [48]. This decision is based on the significant effect that 50% AS has shown in several studies [1, 14, 50] and on the hypothesis that at higher concentrations some components can become harmful. In fact, the concentration of biologically active molecules is different in serum and tear fluids. There are no data on which concentration of AS is most appropriate for treating ocular surface diseases. For example, Gupta et al. [51] report that the TGF-*β* concentration in human serum is approximately 50 ng/mL, which is 5 times higher than the amount in tears, and TGF-*β* has antiproliferative effects and high concentrations of this molecule may suppress wound healing of the epithelium [51, 52]. Jeng and Dupps Jr. [14] say that in their series 100% AS has a very high concentration of serum proteins that could alter the osmolality and pH of the preparation; in addition, patients enjoy the extra viscosity of 50% AS, compared to 20% AS. In addition, we must consider that the frequency of venipuncture and the amount of blood needed are doubled with the use of 100% AS drops [11]. Therefore, even if 100% AS drops were more effective, we believed 50% AS eye drops were safer and more manageable.

The main focus of the study was to show the clinical success of AS eye drops in the treatment of different ocular surface diseases. All collected data were analyzed complexly and subsequently in 2 distinct groups of pathologies—acute eye pathology (i.e., group 1) and chronic eye pathology (i.e., group 2)—with the aim of understanding the potential and the efficacy of this therapy.

The data analysis showed that all patients achieved a significant improvement in symptoms and all patients had excellent compliance with the treatment. No patient in the current study has reported any adverse effects to date; however, some studies have reported adverse effects [39]. In our patients, the treatment was safe and no patients reported allergy intolerance, deposits, or infection.

Best corrected visual acuity improved in all patients, which was in contrast to the study of Ziakas et al. [30] (Figures 3(a) and 3(b)). The average improvement was greater in patients with acute diseases, but this difference occurred because of a higher rate of eye comorbidity, which was also influenced by the age of the patients.

In primary or secondary tear deficiency, the AS supplies the lacking factors and reestablishes a correct ocular surface balance. This is the situation with dry eyes, Sjögren and non-Sjögren-related. For mild dry eyes, artificial tears, when frequently applied, are usually effective since they are able to reduce symptoms and prevent complications and the progression of damage [53, 54]. However, in more severe cases, artificial tears may be unable to stabilize the framework, and the damage to the ocular surface worsens with dramatic consequences such as eye ulcers to eye perforation [55]. In 1984, Fox et al. [7] were the first to report the beneficial effects of applying AS eye drops to dry eye in Sjogren's syndrome. Tsubota et al. [8] later revealed increased numbers of goblet cells and decreased squamous epithelium metaplasia after AS therapy. Compared to patients treated with nonpreserved artificial tears, Kojima et al. [39] found a significant improvement in tear stability, ocular surface vital staining scores, and pain symptom scores in patients treated with AS.

In our study, the patients with dry eye syndrome started therapy with AS eye drops because the symptom had not resolved with conventional therapy and they had a long history of chronic inflammation and recurrent epithelial defects leading to infection, opacity, and visual capacity damage [56]. All patients improved in objective and subjective symptoms. Four patients are currently without AS treatment but maintain artificial tears therapy. They are all followed up regularly and show a stable framework with no recurrence. However, the therapy suspension period remains short and does not allow stating the stability of the framework. It seems that, in cases of medium to serious ocular dryness, AS therapy is able to give stability, even if the duration must be verified.

**Figure 1:**
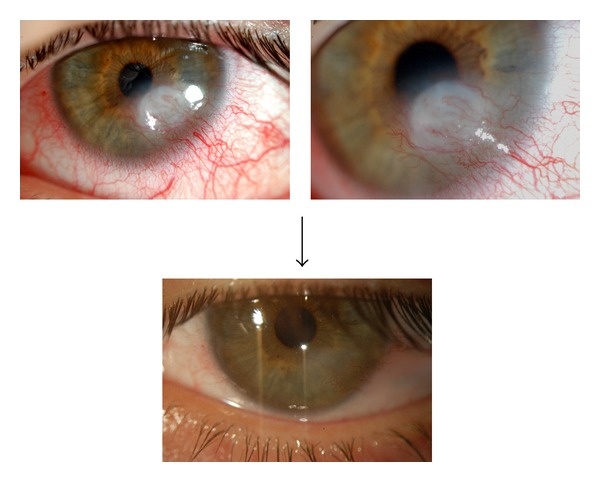
Clinical evolution of a patient with neurotrophic keratitis due to trigeminal nerve injury that occurred during acoustic neuroma excision.

**Figure 2:**
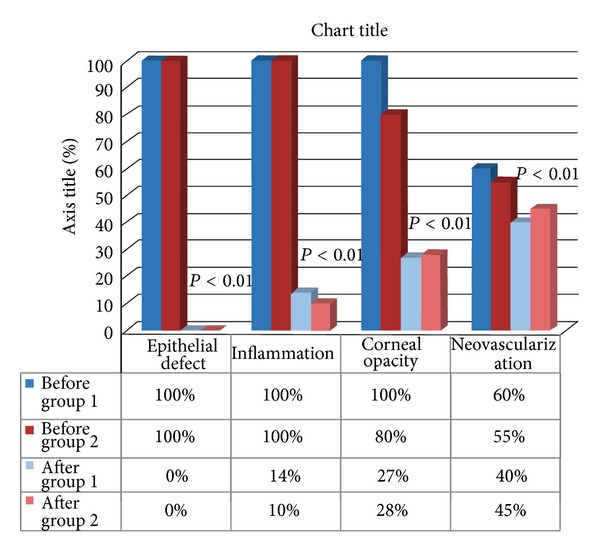
Evolution of clinical signs in group 1 and group 2. All data significantly improved.

**Figure 3:**
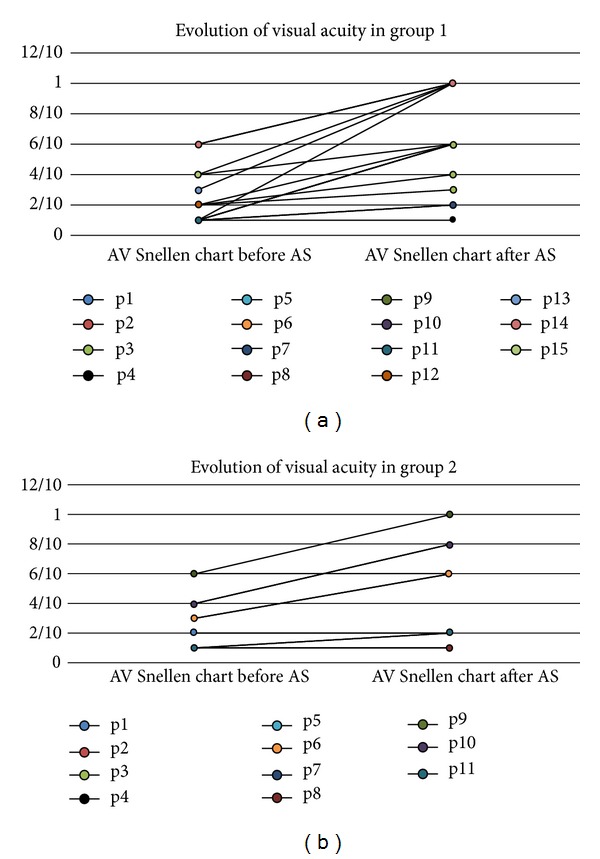
Evolution of visual acuity in the patients in the 2 groups. AS = autologous serum; p1–p15 = patient 1–patient 15.

In neurotropic keratopathy, a different mechanism occurs [57]. In this disease, there is not a single deficiency of tears but rather an imbalance with the production of harmful substance and an increased need for trophic factors. Numerous ocular and systemic diseases may lead to neurotropic keratopathy. In these diseases, neural factors such as acetylcholine or SP are depleted from the cornea. Nishida et al. [41] emphasize the importance of SP and IGF for a normal wound-healing response and Matsumoto et al. [28] report efficiency in the treatment of neurotropic keratopathy with 20% AS eye drops. Matsumoto showed that AS contains nerve growth factor and SP levels that are several times higher than the levels in tears and harbors IGF-1. It is their belief that AS helps healing in neurotropic keratopathy by providing lubrication and nerve healing and epithelialization. In another study, López-García et al. [58] report that, in aniridic keratopathy, AS eye drops improve the ocular surface and give more comfort compared to artificial tears. In our neurotropic keratopathy series patients, 3 patients had postherpetic keratitis, 2 patients had trigeminal nerve injury, and 1 patient had aniridic keratopathy. All patients had long-term therapy with tear substitutes and a history of recurrent trophic ulcers, significant neovascularization, and corneal scarring. On average, these patients had frameworks more severe than previous cases of dry eye, particularly when the deficit involved facial paralysis and anomalies in the dynamics of the eyelids. In all patients, we obtained stabilization of the framework, which was characterized by epithelial stability (i.e., more regular epithelium without defects) and a reduction in neovascularization, inflammation, and corneal opacity (Figure 1). One patient with trigeminal injury remains in therapy after 2 years, whereas the other patient, after discontinuing AS therapy, has relapsed with a trophic ulcer at risk of perforation. This experience allows us to believe that, especially in severe cases of neurotropic damage, the contribution of AS factors should be continuous and a program of chronic therapy can be scheduled.

The patients with ocular burn represent a different framework in which a single traumatic event, the acid burn, leads to an ocular surface injury with an action at term, but persistent damage. Few studies are available in the literature on chemical burns, and used as a treatment PRP [32], umbilical cord serum [33], or amniotic membranes [59]. Corneal chemical burns cause corneal infection, ulceration, opacity, and neovascularization. Irrespective of the source of regenerating epithelium, the rate of migration after chemical injury is reduced [60–63]. Therefore, the primary aims of therapy are the promotion of epithelialization as fast as possible, reduction of inflammation, support of reparative processes, and prevention of complications with the least permanent damage. Subconjunctival autologous regenerative factor-rich plasma in ocular alkali burns is able to appreciably reduce corneal and conjunctival epithelialization time, sick leave duration, and healing time [64]. In a small sample, Panda et al. [32] showed significant improvement in the healing of epithelial defects, corneal clarity, and BCVA with the inoculation of PRP. Márquez-de-Aracena et al. [65] showed a shorter corneal healing time with the use of subconjunctival platelet concentrate autologous injection in comparison to conventional therapy. They also state that it is unnecessary to activate PRP and suggest using it topically [65, 66].

The rationale of using AS in chemical burns derives from the fact that it contains antiproteases such as alpha 2 macroglobulin (which reduces collagenase) and vitamin A (which modulates the normal growth and differentiation of the epithelium) [38, 67]. It modulates the expression of TSP1 to accelerate epithelialization [65] and inhibits VEGF-A [38, 68].

In our casuistry, 5–7 days after injury, all patients had evident signs of corneal suffering with epithelial instability and inflammation that was caused by trophic damage on the cornea and conjunctiva, despite limited limbal deficiency. The study of clinical signs showed that in each patient we attained the primary objective, which was the stabilization of the condition in the absence of inflammation, and a complete restitution of epithelial integrity. The regression of neovascularization in most patients may be because of an increase in trophic factors and a decrease in inflammatory factors. In fact, neovascularization that is formed to provide nourishment disappears quickly if the stimulus ceases. Opacity declined because of the reduction of inflammation and because of stromal remodeling supported by serum factors such as EGF, fibronectin, TGF-*β*, retinoic acid, and nerve growth factor that are able to promote proliferation and differentiation of limbal corneal epithelium cells.

All our patients obtained these results, although with different timings between acute and chronic diseases. Chemical burns had a shorter average treatment time. This suggests that chemical injury is reversible and the ocular surface stability can be self-maintained if inflammation is properly reduced and growth factors are rebalanced. We suggest that our patients had a limited limbal ischemia with a largely preserved limbus. None of the chemically burned patients remain in therapy and all patients have a healthy ocular surface.

A limitation of our study is the lack of a control arm for the acute group patients. A future goal will be to followup with a control arm treated with anti-inflammatory drugs and artificial tears to make direct comparisons between both therapies in chemical injury.

## 5. Conclusions

In our casuistry, AS eye drops have been effective in improving and stabilizing signs and symptoms in patients who do not improve with conventional therapy. We believe that a reconfirmation of our findings will be desirable in a larger group of patients in a prospective controlled trial setting. Studies aimed at clarifying the beneficial effects and risks of prolonged application of AS drops at different AS concentrations should also be the subject of future investigations.

In addition, a future goal will be to conduct examinations, especially in the composition of tears. This can help scientists understand what factors are decreased in the tears of these patients and how they are decreased and how long AS components can effectively remain in tears after therapy.

## Figures and Tables

**Table 1:** Inclusion and exclusion criteria of the study.

Criteria	Inclusion criteria	Exclusion criteria
General		Inability of venous blood sampling because of (i) venous access being not available,, (ii) anemia, (iii) cerebrovascular or cardiovascular disorders, (iv) being positive for viral markers (HBV, HCV, and HIV), (v) being bacterial active, (vi) women who are pregnant or breast-feeding, (vii) patients unable to provide informed consent, (viii) age under 18 years.

Chemical eye burn	(i) Persistent epithelial defect (ii) Inflammation and/or opacity and/or corneal neovascularization	(i) Corneal perforation/melting (ii) Limbal stem cell deficit (iii) Patients with active infections of the eye or eyelid (iv) Abnormalities of the eyelid
Severe dry eye syndrome	(i) Symptoms of dry eye with daily activity limitation (ii) Time to break-up <5 sec (iii) Schirmer's test without anaesthesia <5 mm after 5 minutes (iv) Fluorescein staining positive with or without epithelial defect (v) Patients refractory to conventional therapy	
Neurotrophic keratitis	(i) Patients with persistent epithelial defects (ii) Patients refractory to conventional therapy	

HBV = hepatitis B virus; HCV = hepatitis C virus; HIV = human immunodeficiency virus.

**Table 2:** Summary of the 26 eyes treated with autologous serum eye drops.

Eye	Sex	Age (y)	Etiology	Weeks of serum therapy	Comorbidity
1	M	41	CB	24	None
2	M	22	CB	24	None
3	M	59	CB	12	None
4	F	81	CB	12	Diabetes mellitus
5	M	29	CB	24	None
6	M	41	CB	12	None
7	M	30	CB	48	None
8	M	32	CB	12	None
9	M	38	CB	12	None
10	M	55	CB	12	None
11	M	31	CB	12	None
12	M	50	CB	24	None
13	M	19	CB	24	None
14	M	23	CB	12	None
15	M	43	CB	12	None
16	F	71	SS	24	None
17	F	37	NK	24	s/p PK
18	F	64	NK	12	s/p trabeculectomy
19	M	71	DE	12	None
20	M	80	DE	24	None
21	M	60	NK	12	Trigeminal neuralgia
22	F	79	SS	48	None
23	F	44	NK	48	s/p trabeculectomy
24	M	37	NK	72	Acoustic neuroma
25	F	40	NK	12	None
26	F	51	NK	48	Lagophthalmos

CB = chemical burn; DE = dry eye syndrome; NK = neurotrophic keratopathy; s/p PK = status postpenetrating keratoplasty; s/p trabeculectomy = status posttrabeculectomy; SS = Sjogren's syndrome.

**Table 3:** Summary of the distribution and evolution of the clinical signs in the 2 groups of patients.

Signs	Presence of sign PRE-AS gr 1	Presence of sign POST-AS gr 1	Distribution of grades	Distribution of grades	Average of the grades PRE-AS GR.1	Average of the grades POST-AS GR.1	*P* value
Epithelial defect	100%	0%	GRADE 3: 73%	GRADE 3: 0%	2.73 ± 0.46	1	<0.01
			GRADE 2: 27%	GRADE 2: 0%			
			GRADE 1: 0%	GRADE 1: 100%			

Inflammation	100%	14%	GRADE 3: 73%	GRADE 3: 0%	2.73 ± 0.46	1.13 ± 0.35	<0.01
			GRADE 2: 27%	GRADE 2: 13%			
			GRADE 1: 0%	GRADE 1: 87%			

Corneal opacity	100%	27%	GRADE 3: 84%	GRADE 3: 0%	2.93 ± 0.26	1.40 ± 0.51	<0.01
			GRADE 2: 6%	GRADE 2: 40%			
			GRADE 1: 0%	GRADE 1: 60%			

Neovascularization	60%	40%	GRADE 3: 33%	GRADE 3: 0%	1.93 ± 0.88	1.20 ± 0.41	<0.01
			GRADE 2: 27%	GRADE 2: 27%			
			GRADE 1: 40%	GRADE 1: 73%			
Epithelial defect	100%	0%	GRADE 3: 73%	GRADE 3: 0%	2.73 ± 0.46	1	<0.01
			GRADE 2: 27%	GRADE 2: 0%			
			GRADE 1: 0%	GRADE 1: 100%			

Inflammation	100%	10%	GRADE 3: 64%	GRADE 3: 0%	2.64 ± 0.51	1.09 ± 0.30	<0.01
			GRADE 2: 36%	GRADE 2: 19%			
			GRADE 1: 0%	GRADE 1: 81%			

Corneal opacity	88%	28%	GRADE 3: 45%	GRADE 3: 0%	2.27 ± 0.79	1.27 ± 0.47	<0.01
			GRADE 2: 36%	GRADE 2: 45%			
			GRADE 1: 19%	GRADE 1: 55%			

Neovascularization	55%	45%	GRADE 3: 36%	GRADE 3: 0%	1.67 ± 0.52	1.18 ± 0.40	<0.01
			GRADE 2: 18%	GRADE 2: 27%			
			GRADE 1: 46%	GRADE 1: 73%			

Post-AS = postautologous serum treatment; pre-AS = preautologous serum treatment.
